# Value of caveolin-1 in cancer progression and prognosis: Emphasis on cancer-associated fibroblasts, human cancer cells and mechanism of caveolin-1 expression (Review)

**DOI:** 10.3892/ol.2014.2385

**Published:** 2014-07-28

**Authors:** DALI CHEN, GUOWEI CHE

**Affiliations:** Department of Thoracic Surgery, West China Hospital, Sichuan University, Chengdu, Sichuan 610041, P.R. China

**Keywords:** caveolin-1, cancer-associated fibroblasts, mechanisms, human cancer cells, cancer progression

## Abstract

Caveolin-1 (Cav-1) is found predominately in terminally differentiated cells, such as adipocytes, endothelia and smooth muscle cells, as well as type I pneumocytes. As a main structural component of caveolae, Cav-1 is important in modulating cellular signaling. In the present study, the expression and clinical role of Cav-1 were analyzed in tumor stromal and human cancer cells, respectively. The results of previous studies have shown that the downregulation of tumor stromal Cav-1 promotes tumor survival and predicts a poor tumor prognosis, predominantly concentrating on the mechanism of the metabolism of the cancer microenvironment (according to the autophagic tumor stroma model of cancer metabolism and the reverse Warburg effect). However, contradictory results concerning the expression, clinical roles and associated mechanisms of Cav-1 have been reported. An improved understanding of Cav-1 expression in tumor stromal and cancer cells will increase knowledge with regard to the clinical value of Cav-1 and its detailed mechanisms. This review summarizes the novel data concerning the clinical values and probable mechanisms of Cav-1 expression in tumor stromal (predominantly in cancer-associated fibroblasts) and cancer cells, respectively.

## 1. Discovery and function of caveolin (Cav)-1

The identification of Cav-1 began with a study into the morphological observations of the caveolae in the 1950s. Caveolae are morphologically identifiable plasma membrane invaginations that can be identified by electron microscopy; caveolae, which are 50–100 nm in size (1), appear as vesicles at the plasma membrane. Cav (now termed Cav-1) was initially identified as one of the four major proteins to be resistant to extraction with non-ionic detergents and to demonstrate a staining pattern in Rous sarcoma-transformed chicken embryonic fibroblasts (2). Cav-2 and Cav-3 were subsequently identified through various experiments (3,4). Further study has indicated that Cav-1 expression is sufficient and necessary to drive the formation of morphologically identifiable caveolae (5), making it the first true protein marker of caveolae (6). To date, three members of the Cav gene family have been identified. The gene locus of Cav-1 is on human chromosome 7q31.1, located adjacent to Cav-2 (~19 kb apart), while Cav-3 is located on a different chromosome (3p25) (7). Two study groups (8,9) independently revealed that Cav-1 is identical to the vesicular integral-membrane protein of 21 kDa and, subsequently, was confirmed to be the same protein.

As the first member of the Cav family (including Cav-1,-2 and -3), Cav-1, a 22-kDa protein of 178 amino acids, has been the most sufficiently investigated by a number of biochemical studies. Cavs are found predominantly at the plasma membrane, however, their expression levels vary considerably between tissues. The highest levels of Cav-1 are found in the terminally differentiated cells, such as adipocyte, endothelia and smooth muscle cells, as well as type I pneumocytes. The localization and expression of Cav-2 is mapped to Cav-1 and is required for the proper membrane localization of Cav-2, whereas Cav-3 is expressed predominantly in the muscle cells, including the smooth, skeletal and cardiac myocyte cells (4).

It was initially identified that Cav-1 is resistant to extraction with sodium carbonate and high salt concentrations, which demonstrated that it is an integral membrane protein (6). It has been suggested that the amino and carboxyl termini of Cav-1 face the cytoplasm, with a hydrophobic domain inserted into the membrane via the classical endoplasmic reticulum machinery. The membrane insertion (residues 102–134) are considered to form a unique hairpin loop configuration that prevents Cav-1 from completely spanning the plasma membrane in a traditional double-pass fashion (9). Mutational analysis and domain mapping experiments have demonstrated the importance of two other regions of Cav-1 that bind to membrane with high affinity (11–14). These regions are now known as the NH_2_-terminal membrane attachment domain (N-MAD; residues 82–101) and COOH-terminal membrane attachment domain (residues 135–150). The oligomerization domain (residues 61–101) of Cav-1 meditates the homo-oligomerization of 14–16 Cav-1 isoforms (15), which subsequently form high molecular mass oligomers of ~400 kDa through several stages of oligomerization. The N-MAD (residues 82–101) are also termed the Cav scaffolding domain (CSD). Couet *et al* (16) identified two Cav binding motifs (CBMs; ϕχϕχχϕ and ϕχχχχϕχχ, where ϕ represents an aromatic amino acid and χ represents a non-aromatic amino acid) in the majority of proteins matched to the CSD of Cav-1. Via an interaction between the CSD of Cav-1 and Cav binding domain. of a given caveolae-associated protein, a number of specific signaling molecules may be concentrated and regulated by Cav-1, including G-protein subunits, receptor or non-receptor tyrosine kinases, and endothelial nitric oxide (NO) synthase (NOS). As a scaffolding protein, Cav-1 also serves as a signal transduction molecule that inhibits or enhances the signal activity of a given caveolae-associated protein. Furthermore, numerous studies have suggested that Cav-1 serves as a negative regulator of cell proliferation or as a tumor suppressor.

## 2. Cav-1 and cancer-associated fibroblasts (CAFs)

### Prognostic value of the downregulation of stromal Cav-1 expression

To date, the conception of tumorigenesis has been exclusively focused on the transformation of cancer cells themselves to the complex cross-talk between cancer cells and the tumor microenvironment. Furthermore, CAFs constitute a major portion of the tumor microenvironmental elements, including the extracellular matrix (ECM) (1), pericytes (2), endothelial cells, immune and inflammatory cells (5) and secreted diffusible growth factors/cytokines (17). A number of studies have suggested that CAFs are key in cancer progression. CAFs retain a major role in ECM remodeling that has been reported to influence the proliferation, survival and migration of cancer cells (18–21). In addition, activated CAFs secrete components including collagen types I and IV, extra domain A-fibronectin, hepatocyte growth factor, epidermal growth factor, basic fibroblast growth factor, extracellular matrix components and matrix metalloproteinases (22,23). CAFs also show an ability to prevent cancer cell apoptosis and induce the proliferation of surrounding cancer cells. Compared with normal fibroblasts (NFs) mixed with epithelial tumor cells, CAFs are more accomplished at enhancing tumor growth and give rise to highly vascularized tumors. According to associated studies (24–26), certain biological molecules can be recognized as biomarkers of CAFs, including α-smooth muscle actin, fibroblast-specific protein 1, fibroblast activation protein and PDGFRα/β. However, there is little knowledge regarding the origin of CAFs and the mechanisms of phenotype transformation from benign to heterogeneous fibroblasts (such as CAFs).

As a principal component of the protein coat of caveolae, Koleske *et al* (27) observed that the level of Cav was clearly reduced in a NIH 3T3 cell line transformed by the expression of the v-abl, bcr-abl, H-ras, polyomavirus middle T antigen or crkl oncogenes, and suggested that the deregulation of Cav (now termed Cav-1) may promote oncogenic transformation. However, Cav-1 has been found to be expressed in the plasma membrane of various types of differentiated cells. The downregulation of Cav-1 is a major characteristic of CAFs and existing studies have indicated that CAFs have the ability to prevent cancer cell apoptosis, enhance the proliferation of cancer cells and stimulate tumor angiogenesis. It is also implicated that the downregulation of Cav-1 is one of the mechanisms that mediates the transformation of fibroblasts.

The majority of these studies have concentrated on breast CAFs. Mercier *et al* (28) were the first to demonstrate that the Cav-1 protein is downregulated in human breast cancer (eight out of 11 patients showed a marked downregulation of Cav-1 protein expression in CAFs by western blot analysis), and observed that CAFs are more numerous in human breast cancer, with an elongated appearance and hyperproliferative when compared with NFs, suggestive of a transformed phenotype. The treatment of Cav-1-deficient CAFs with a Cav-1 mimetic peptide can reverse the hyperproliferation phenotype with a three-fold reduction. Sotgia *et al* (29) further established a direct cause-effect association between stromal Cav-1-deficient and CAF phenotypes, by creating a Cav-1(−/−) mammary stromal fibroblast (MSF) cell line. The authors showed that Cav-1(−/−) MSFs share a number of properties with human CAFs, including similar gene profiles, the functional inactivation of the retinoblastoma (RB) tumor suppressor and the functional characteristics of myofibroblasts. Such initial discoveries lead to the proposal that Cav-1 may serve as a cancer prognostic factor. Sloan *et al* (30) further analyzed tissue sections specifically for the stromal and tumor epithelial cell expression of Cav-1 from two cohorts of breast cancer patients. In total, 103 out of 173 patients (60%) and 31 out of 429 patients (7%) exhibited unambiguous staining for the stromal compartment of the tumor and epithelial tumor cells, respectively. According to El-Gendi *et al* and Witkiewicz *et al* (31,32), the presence of Cav-1 in epithelial tumor cells positively correlates with lower TNM tumor stage (P=0.05), but does not predict the cancer-specific survival of more than five years. By contrast, the loss of stromal Cav-1 has been found to positively correlate with the previously described clinical characteristics of breast cancer (30). Together with Witkiewicz *et al* (33), these studies separately revealed an independent prognostic value of the downregulation of breast tumor stromal Cav-1. The 10-year survival rate for patients with tumors positive for Cav-1 expression in the stroma is 91%, when compared with 43% for patients lacking stromal Cav-1 (P=0.0001) (30). Similarly, the loss of stromal Cav-1 expression predicts poor clinical outcome in triple negative and basal-like breast cancers (32). The overall survival rate has also been found to decrease with the deregulation of tumor stromal Cav-1. Notably, TN patients with high-levels of stromal Cav-1 have a good clinical outcome, with >50% of the patients surviving the follow-up period. By contrast, the median survival time for TN patients with moderate stromal Cav-1 staining is 33.5 months. Similarly, the median survival time for TN patients with absent stromal Cav-1 staining is 25.7 months (32). In a combined study of 358 resected breast cancers cocultured with Cav-1 siRNA-treated fibroblasts and the MDA-MB-468 cell line, Simpkins *et al* (34) identified that the loss of Cav-1 expression significantly correlates with decreased breast cancer-specific and disease-free survival (P=0.01), through promotion of breast cancer cell invasion. Overall, this clinicopathological study of breast cancer revealed a significant correlation between the absence of stromal Cav-1, and larger tumor size, advanced tumor stage (TNM stage), higher grade, lymph node metastasis, poor tumor prognosis and short overall survival time.

Similar clinical values of decreased stromal Cav-1 levels have also been found in gastric cancer (GC) (35,36), prostate cancer (PC) (37,38) and malignant melanoma (39). Additionally, Zhao *et al* (35) found that positive rates of epithelial Cav-1 expression in gastritis without intestinal metaplasia (IM), gastritis with IM and GC showed a decreasing trend compared with gastritis without IM, gastritis with IM and GC (P=0.012). Furthermore, no significant correlation was identified between tumor cells and CAF Cav-1 expression (P=0.751). The expression of Cav-1 in CAFs was also found to significantly correlate with disease-free survival (P=0.029) and overall survival (P=0.013) (35). In addition, the downregulation of stromal Cav-1 was found to predict poor survival, early recurrence and a lower cumulative five-year survival rate for GC patients. Furthermore, multivariate analysis (COX proportional-hazard regression model) revealed (35) that CAF Cav-1 expression is an independent predictor of recurrence and survival in GC patients, consistent with the study by He *et al* (36). No correlation has been identified between the expression stature of stromal Cav-1 and the typical clinicopathological parameters of GC, such as T stage, TNM stage and Lauren classification. Notably, no correlation has been identified between Cav-1 expression in tumor cells, and the prognosis and clinicopathological parameters of GC. The decreased trend of stromal Cav-1 in patients with benign prostatic hypertrophy, primary PCs and PC metastases have also been identified (38). Furthermore, a large cohort of 724 PC patients demonstrated a significant correlation between decreased levels of stromal Cav-1, and increased Gleason score (P=0.012) and reduced relapse-free survival (P=0.009) (37). Studies (37,38) have also found a correlation between the loss of stromal Cav-1 and upregulation of Akt phosphorylation, suggesting that the loss of Cav-1 in the tumor microenvironment contributes to the metastatic behavior of tumor cells by a mechanism that involves the upregulation of transforming growth factor (TGF)-β1 and SNCG through Akt activation. In malignant melanoma, the positive correlation between the loss of stromal Cav-1 and poor overall survival rate has been clarified by Wu *et al* (39). The authors identified that a low stromal Cav-1 expression correlates with shorter survival when compared with the high stromal Cav-1 expression group (median survival, 252 days vs. 3,508 days, respectively; P=0.0054).

### Conceivable mechanisms

**Figure 1:**
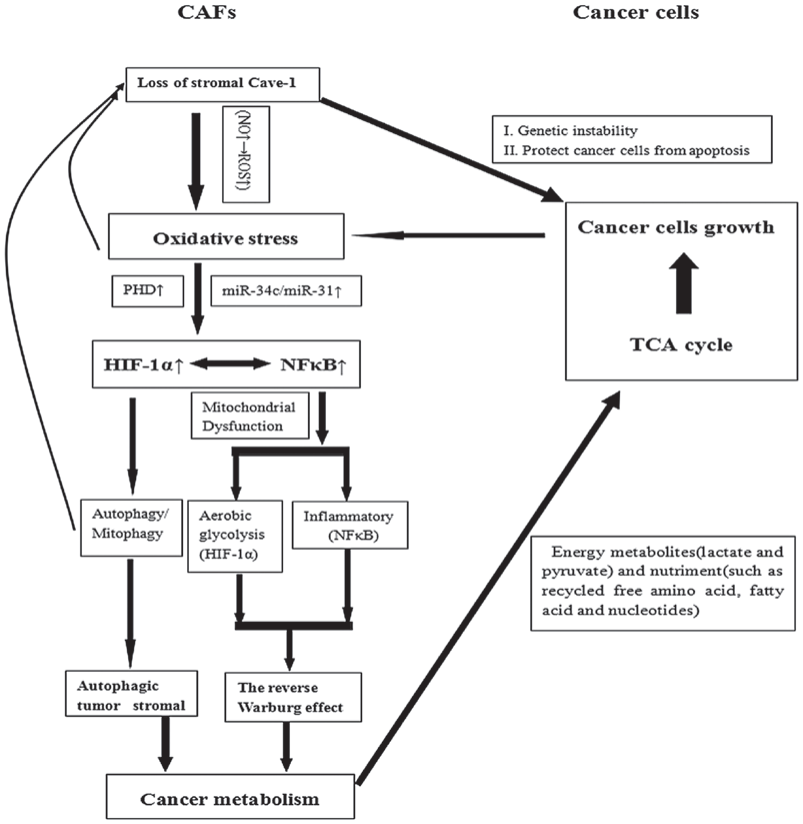
Conceivable mechanisms: Cav-1-deficient CAFs predict tumor prognosis through the autophagic tumor stroma model of cancer metabolism and the reverse Warburg effect. CAFs, cancer-associated fibroblasts; TCA, tricarboxylic acid cycle; Cav-1, caveolin-1; PHD, prolyl hydroxylase domain-containing protein; HIF-1α, hypoxia inducible factor-1α; NFκB, nuclear factor κB; NO, nitric oxide; ROS, reactive oxygen species.

Cav-1-deficient CAFs may predict tumor prognosis (Fig. 1). Numerous studies (28,29,40,41) support that the deregulation of stromal Cav-1 serves as a functional marker of CAF phenotype. Sotgia *et al* (29) established a Cav-1(−/−) MSF cell line that shares numerous characteristics with human CAFs, such as an almost identical profile of RB/E2F-regulated genes that are upregulated in human CAFs. The phenotype of the Cav-1-deficient CAFs may be reversed by treatment with a Cav-1 mimetic peptide (28). According to these previous studies, we hypothesize at least three possible mechanisms of Cav-1 deregulation as follows. Firstly, the activation of oncogenes (H-ras, v-abl, brc-abl and TGF) or inactivation of tumor suppress genes (p53) may result in a loss of Cav-1 expression in CAFs in culture (42). Secondly, similar to the activation of fibroblasts in wound healing, the activation of the TGF-β signaling pathway may also result in the Cav-1 deregulation of CAFs. Furthermore, CAFs have been shown to secrete a number of growth factors, including TGFβ (43). Finally, surrounding cancer cells may downregulate Cav-1 in adjacent NFs via oxidative stress to the tumor microenvironment (40).

Martinez-Outschoorn *et al* (44) observed that the coculture of immortalized human fibroblasts or primary cultures of normal human fibroblasts with a human breast cancer cell line (MCF7) leads to Cav-1 downregulation in fibroblasts, acquiring a CAF phenotype. In addition, the transcription levels of Cav-1 in CAFs have been found to increase by 2.3- to 2.4-fold or remain unchanged (28), suggesting that the downregulation of stromal Cav-1 occurs at the post-transcriptional or -translational level. In accordance with similar studies, we suggest that the prognostic value of the downregulation of the stromal Cav-1 is predominantly associated with the metabolism of the tumor microenvironment, which is carefully discussed in the present review, including the autophagic tumor stroma model of cancer metabolism and the reverse Warburg effect. In addition to the induction of the hyperproliferative phenotype of CAFs through the RB/E2F pathway.

Several studies have indicated that the decrease of stromal Cav-1 is accompanied by the activation of the RB/E2F pathway (28,33). Using the gene profiling method, the authors identified 118 gene transcripts involved in cell cycle control that were upregulated. Among them, 44 gene transcripts were involved in the RB/E2F gene signature, associated with RB functional inactivation. RB is normally hypophosphorylated in quiescent or differentiated cells, and prevents the transcription of genes essential for cell cycle progression by suppressing the activity of the E2F family (45). The downregulation of stromal Cav-1 upregulates the phosphorylation of RB and releases the activity of E2F, increasing downstream target molecules, such as proliferating cell nuclear antigen (PCNA) and minichromosome maintenance protein (MCM7), which account for the hyperproliferative phenotype of CAFs. PCNA is a transcription factor which helps the DNA polymerase δ to bind to the DNA, while MCM7 serves as an inhibitor of DNA replication when bound to hypophosphorylated RB. In addition, certain reports have prompted that RB is a downstream molecule of mTOR in adipocytes, prostate and ovarian cancer cells (46–48). Although other reports have found that mTOR is activated in Cav-1 knock out CAFs (49) and keloids (50), the newly identified axis in CAFs (Cav-1 to mTOR to RB) (49) requires further clarification.

### Autophagic tumor stroma model of cancer metabolism

The theory of ‘the autophagic tumor stroma model of cancer metabolism’ is a newly established model to understand the prognostic value of the downregulation of stromal Cav-1. Martinez-Outschoorn *et al* (51) initially demonstrated this new paradigm, which further confirmed the ‘autophagy paradox’ that the role of autophagy in tumorigenesis is controversial. Mechanistically, the authors also demonstrated that the state of oxidative stress in adjacent CAFs results in the autophagic/lysosomal deregulation of stromal Cav-1 via elevated hypoxia inducible factor (HIF)-1α and nuclear factor κB (NFκB) (44,52). Therefore, a positive feedback exists between oxidative stress and the loss of stromal Cav-1. Although the detailed mechanisms in which the loss of stromal Cav-1 causes oxidative stress remain undistinguishable, this review summarizes a possible mechanism based on previous studies.

As a potent inhibitor of NOS, Cav-1 binds to and inhibits NOS activity in NFs, thus dampening NO release in a toxic manner (53). However, studies (41,54) have found that NOS productions are transcriptionally overexpressed in human tumor and Cav-1(−/−) stromal cells. This indicates that the loss of stromal Cav-1 is deprived of its ability to inhibit NOS activity and to induce the overexpression of NO. Besides resulting in DNA damage, the accumulation of NO also induces mitochondrial uncoupling and increased reactive oxygen species (ROS). As the mitochondrial respiratory chain is a major source of intracellular ROS. The mitochondrial uncoupling induced by the overexpression of NO results in the dysfunction of the mitochondrial respiratory chain and largely increases the levels of ROS. Finally, oxidative stress is generated with the upregulation of ROS. Concomitantly, it has been identified that the subunits of the respiratory chain complexes (complex I, IV and V) are significantly decreased in Cav-1 knockdown fibroblasts, and ROS is markedly upregulated in human telomerase reverse transcriptase (hTERT)-fibroblasts treated with Cav-1 siRNA (52). Notably, compared with the homotypic cultures of fibroblasts, ROS levels are significantly increased in fibroblasts (immortalized with hTERT) when cocultured with human breast cancer cells (MCF7). Taken together, the downregulation of Cav-1 and ROS levels are under ‘positive feed-forward control’ in CAFs, where an accumulation of ROS induces the downregulation of stromal Cav-1, which results in the subsequent generation of ROS (52).

The state of oxidative stress in the tumor microenvironment is triggered by lateral epithelial cancer cells and sustained through positive feed-forward control with the downregulation of stromal Cav-1. Studies have already demonstrated stromal oxidative stress based on the methods of proteomic and/or transcriptional gene profiling. Witkiewicz *et al* (40) identified the upregulation of 238 gene transcripts and the downregulation of 232 gene transcripts in the Cav-1-deficient tumor stroma. The gene set enrichment analysis illustrated that the upregulation of gene transcripts is associated with myofibroblast differentiation, oxidative stress, mitochondrial dysfunction and DNA damage. Trimmer *et al* (55) also identified the upregulation of 21 gene products associated with oxidative stress and hypoxia, including glycolytic enzymes (LDHA and GAPDH), mitochondrial components involved in ROS production, enzymes acting as antioxidants (PRDX1, PRDX4 and TXNDC5) and factors that are involved in oxidative stress-induced DNA repair (XRCC6BP1). Pavlides *et al* (54) provided evidence of stromal oxidative stress, having identified ~100 metabolites (the two most significant metabolites being asymmetric dimethylarginine and 3-hydroxybutyrate), which were associated with the onset of the oxidative stress phenotype, that were elevated in Cav-1 (−/−) null mammary fat pads.

Studies have demonstrated that oxidative stress in adjacent CAFs induced by epithelial cancer cells result in autophagy/mitophagy in the tumor microenvironment (44,51,52,54). Combined with the stromal oxidative stress, studies have also identified the upregulation of numerous molecules in adjacent CAFs, which have been specifically associated with autophagy/mitophagy, as well as mitochondrial dysfunction (40,54,55). In addition, a previous study observed that the expression of autophagy markers is markedly elevated following acute Cav-1 knockdown in fibroblasts, including Beclin 1, BNIP3, BNIP3L, HIF-1α and NFκB (44). This observation demonstrated that the loss of stromal Cav-1 is sufficient to induce autophagy/mitophagy in CAFs. Given that the downregulation of stromal Cav-1 results in autophagy, which is induced by the oxidative stress and hypoxia of the tumor microenvironment, this indicates that a feed-forward mechanism exists in the interactive association between Cav-1 and autophagy/mitophagy in CAFs.

Mechanically, studies have demonstrated that oxidative stress drives autophagy/mitophagy via the meditated induction of HIF-1α and NFκB activation in fibroblasts (44,51,56,57). Therefore, HIF-1α, which is the main transcription factor mediating the hypoxia response, promotes transcription of angiogenic factors [such as vascular endothelial growth factor (VEGF)] and leads to increased autophagy and glycolysis (58). The state of oxidative stress implies the accumulation of ROS in CAFs. In addition, the activation of HIF-1α and NFκB require the reduced activation of prolyl hydroxylase domain-containing protein (PHD). The results of a previous study (52) has shown that Cav-1 knockdown decreases the levels of E1α, E1β and E2 subunits of the PHD complex. Therefore, the reduced activity of PHD mediated by increased ROS levels results in the reduced hydroxylation of HIF-1α, leading to HIF-1α stabilization and activation (59–62). Additionally, the transcriptional activation of HIF1a by miR-31 is indirectly mediated by FIH-1 (factor inhibiting HIF), which is the direct target of miR-31 (54). Capparelli *et al* (57) demonstrated that the activation of the TGFβ/CTGF pathway also regulates the metabolism of CAFs via the elevation of HIF-1α. Additionally, as a multimeric inducible transcription factor, the activation of NFκB is determined by the IκB kinase (IκBK) activity, which is controlled by oxygen-sensitive PHD. The activation of IκBK induced by the downregulation of PHD meditates the deregulation of inhibitor of κB (IκB) by phosphorylation. As NFκB subunits are inhibited and sequestered in the cytoplasm by IκB, the deregulation of IκB meditates the activation of NFκB. Furthermore, recent evidence has demonstrated important cross-talk and the interdependence of HIF-1α and NFκB signaling. Increased HIF-1α has also been shown to promote NFκB activity (63). Conversely, NFκB acts as a transcriptional factor of HIF-1α (64).

### The reverse Warburg effect

The reverse Warburg effect is an additional model that has been proposed to understand the Warburg effect in tumor metabolism. The Warburg effect, known as aerobic glycolysis, was first formulated by Warburg (65). Warburg’s original study demonstrated the propensity of cancer cells to take up high levels of glucose and to secrete lactate and pyruvate (energy metabolites generated by aerobic glycolysis). Furthermore, recent evidence has demonstrated that tumor stromal fibroblasts also exhibit the Warburg effect and secrete energy metabolites (including lactate and pyruvate). In addition, CAFs directly feed cancer cells via a type of host-parasite association. The state of oxidative stress in Cav-1-deficient tumor stroma induced by adjacent tumor cells not only results in autophagy/mitophagy and DNA damage, but also causes mitochondrial dysfunction and aerobic glycolysis (the Warburg effect) (66,67), which is important for cancer recurrence, lymph node metastasis and tumor prognosis. Pavlides *et al* (66) also demonstrated a cause-effect association between the downregulation of stromal Cav-1 and aerobic glycolysis, the upregulation of the myofibroblast marker and eight glycolytic enzymes (including the M2-isoform of pyruvate kinase, as well as HIF target genes) via unbiased proteomic analysis and the transcriptional profiling of Cav-1-deficient stromal cells. This indicated that a loss of stromal Cav-1 may be a novel biomarker for aerobic glycolysis (the Warburg effect) in the tumor microenvironment.

To fully understand the mechanism of the reverse Warburg effect, Pavlides *et al* (41) performed an unbiased informatics analysis of the transcriptional profile of Cav-1(−/−)-deficient mesenchymal stromal cells. The authors identified that Cav-1-deficient stromal fibroblastic cells show a markedly reduced mitochondrial reserve capacity and a mitochondrial defect in Cav-1-deficient stromal cells which may drive oxidative stress, leading to aerobic glycolysis (HIF-1α) and inflammation (NFκB) in the tumor microenvironment. The authors also found that genes associated with NOS production, complexes I and IV and the generation of ROS were upregulated. However, western blot analysis (52) showed a significant decrease in the subunits of complexes (I, IV and V) in Cav-1 knockdown fibroblasts. This contradiction may imply that the loss of mitochondrial respiratory chain complexes occurs at the post-transcriptional or -translational level, or that the upregulation of associated genes is a compensatory response to mitochondrial dysfunction. Concomitantly, fibroblast-MCF7 cocultures, where Cav-1 is downregulated in fibroblasts, show a marked decrease in mitochondrial mass compared with monocultured fibroblasts (52). Further study demonstrated that 45 known HIF-target genes and 86 NFκB-target genes were transcriptionally upregulated in Cav-1(−/−) stromal cells (41). In addition, the upregulation of 151 mitochondrial associated genes served as a compensatory response to mitochondrial dysfunction in Cav-1(−/−) stromal cells. Furthermore, animal experiments have demonstrated that Cav-1-deficient mice suffer from a reduced mitochondrial reserve capacity, and that the lethality of Cav-1 deficiency may be rescued if Cav-1 null mice are fed glucose (68). Overall, we propose a possible mechanism (Fig. 1) to summarize the reverse Warburg effect in tumor stromal fibroblasts.

Several recent studies have markedly suggested that mitochondrial activity and oxidative phosphorylation is sufficient to promote tumor growth. *In vitro* study has shown that MCF7 (human breast cancer cells) cells exhibit extremely high levels of mitochondrial staining when cocultured with Cav-1 null fibroblasts, as compared with homotypic cultures of MCF7 cells (52). Furthermore, lactate administration was found to significantly increase mitochondrial mass in MCF7 cells. This study demonstrated *in vitro* that CAFs undergoing aerobic glycolysis generate and secrete lactate and pyruvate, enhance the mitochondrial respiratory and TCA cycles, and promote tumor growth. By contrast, loss of function mutations in the TCA cycle gene, isocitrate dehydrogenase, are found to correlate with an improved prognosis and survival, suggesting that inactivity of TCA cycle enzymes does not favor tumor aggressiveness (69). The mitochondrial protein, p32, has also been found to maintain high levels of mitochondrial oxidative phosphorylation in human cancer cells and to sustain tumorigenicity *in vivo* (70).

Overall, cancer cells trigger oxidative stress in the tumor microenvironment and activate two pro-autophagic promoters, HIF-1α and NFκB, in stromal CAFs. As a result, adjacent stromal fibroblasts undergo autophagy and mitophagy, leading to the autophagic loss of Cav-1 and mitochondrial dysfunction. A loss of stromal Cav-1 aggravates oxidative stress and further promotes autophagy and mitophagy. As a result, stromal aerobic glycolysis and autophagy/mitophagy generate energy metabolites (lactate and pyruvate) and building blocks (such as recycled free amino acid, fatty acid and nucleotides), respectively, that directly utilize adjacent cancer cells to sustain growth and maintain cell viability.

## 3. The role of Cav-1 expression in human cancer cells

### Expression of Cav-1 in human cancer cells

Cav-1 expression in human cancer cells is not considered to conform with that in the tumor stroma. Therefore, contradictory roles of Cav-1 expression in human cancer cells have been reported. Certain studies insist that Cav-1 is downregulated and serves as a tumor suppressor in breast cancer (71–73), GC (74), hepatic cancer (75) and mucoepidermoid carcinoma (MEC) of the salivary glands (76); while other studies suggest that the expression levels of Cav-1 are upregulated, consistent with advanced tumor stage, high histological type and the metastasis of human cancer cells, including esophagus (77,78), pancreatic (79), renal (80), prostate (81) and colorectal (82) cancer.

The current review of previous studies lead to the recognition of a contradictory theory with regard to the expression of Cav-1 in breast (71–73,83), gastric (74,84), hepatic (75,85,86) and oral (76,87) cancer. Sagara *et al* (73) examined the mRNA and protein expression levels of Cav-1 in 162 cases of breast cancer and found that the mRNA and protein expression levels of Cav-1 were suppressed in breast cancer tissue compared with the corresponding normal tissues. In addition, reduced Cav-1 was found to significantly (P=0.041) correlate with tumor size, consistent with other studies (71,72) However, Savage *et al* (83) questioned the tumor suppressive effect of Cav-1 following the immunohistochemical analysis of Cav-1 expression levels in benign lesions, breast cancer precursors and metaplastic breast carcinomas, in a cohort of 245 invasive breast carcinomas, and a CAV1 gene amplification assessment of 25 cases. Despite its variable intensity, Cav-1 was consistently expressed in MECs of radial scar, sclerosing adenosis, columnar cell lesions and ductal carcinoma *in situ*, and significantly associated with the ‘basal-like’ immunophenotype, with shorter disease-free and overall survival (83). In GC, a study (84) found that the positive staining of Cav-1 was higher in the advance GC group than in the early GC group (P=0.037), whereas, the progressive downregulation of Cav-1 in gastric epithelial cells was found to correlate with gastric carcinogenesis (74). Additionally, Yan *et al* (78) identified that the Cav-1 mRNA expression in hepatitis B virus-related hepatocellular carcinoma (HCC) cells was found to negatively correlate with the tumor size, major venous invasion, single or multiple tumors, pTNM staging and factors associated with the prognosis of HCC, inconsistent with other studies (85,86). Given the conflicting information on the expression of Cav-1, at least in breast cancer, GC, hepatic cancer and oral cancer, further studies analyzing the expression of Cav-1 in human cancer cells are warranted.

In addition, pancreatic (87), esophagus (77), renal (89) and oral (87) cancer have shown the downregulation of Cav-1 in cancer cells compared with non-cancerous tissues. By contrast, breast (83), ovarian (90), hepatic (75) and lung (91,92) cancer exhibit an upregulation of Cav-1 in cancer cells compared with the non-cancerous tissues. This inconsistent phenomenon may be associated with the cell type-related expression of Cav-1; however, further experiments are required to demonstrate the mechanism of the different Cav-1 expression trends in different types of tissue.

### Clinical value of Cav-1 expression in a variety of human cancer types

**Figure 2:**
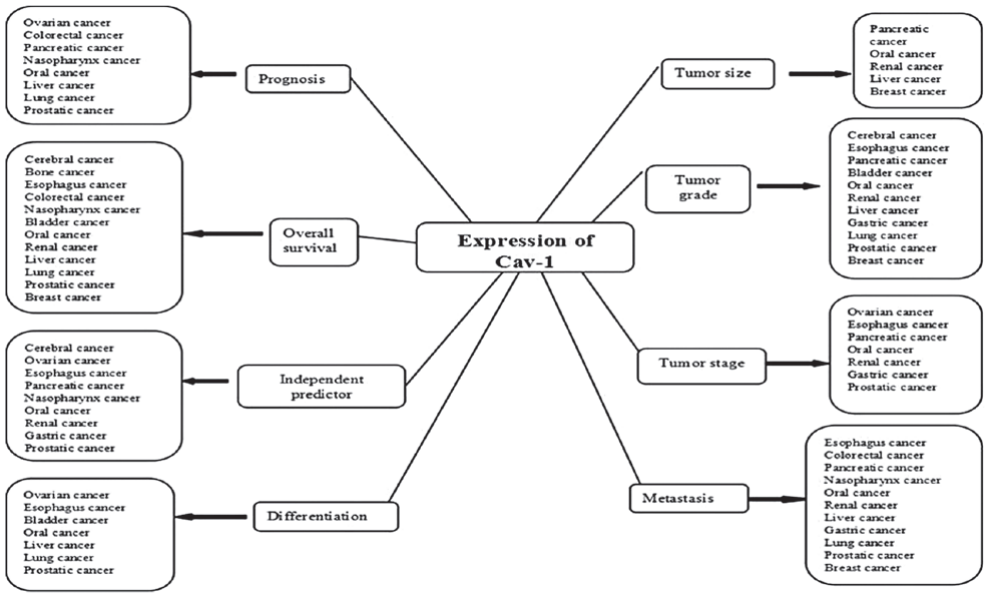
Correlation between the expression of Cav-1 in various types of cancer cells and the clarification of clinical characteristics, including tumor size, differentiation, tumor grade, tumor stage, hematogenous or lymph node metastasis, tumor prognosis and overall survival ratio. Cav-1, caveolin-1.

Despite the contradictory views of the clinical role of Cav-1 expression in several types of cancer, the upregulation of Cav-1 in human cancer cells serves as a tumor promoter role in the majority of human cancer types. The correlation between the expression of Cav-1 in various types of cancer cells and clinical characteristics, including tumor size, differentiation, tumor grade, tumor stage, hematogenous or lymph node metastasis, tumor prognosis and overall survival rate, has been clarified (Fig. 2).

In order to target the clinical value of Cav-1 expression in human cancer cells, this study reviewed the progress of present clinical studies on several types of human cancer.

#### Pancreatic cancer

In pancreatic cancer, Cav-1 is frequently expressed in the tumor tissue compared with the little or no staining identified in chronic pancreatitis specimens, normal ductal epithelium (88) and peritumoral tissue (79,93). The prognostic significance of Cav-1 expression in pancreatic carcinoma was initially demonstrated by Suzuoki *et al* (88). The authors found 32 cases among 79 patients (40.5%) with pancreatic adenocarcinoma showing positive Cav-1 immunostaining. Positive Cav-1 expression was found to correlate with tumor diameter (P=0.0079), histopathological grade (P=0.0272) and poor prognosis (P=0.0008). Finally, the authors suggested that positive Cav-1 expression is an independent negative predictor of survival (P=0.0358). In a recent study of 34 pancreatic ductal adenocarcinoma (PDAC) tissue samples, Tanase *et al* (79) confirmed the prognostic role of the expression of Cav-1. Furthermore, the expression of Cav-1 was found to significantly correlate with Ki-67 and p53, as well as serum levels of CA 19-9. Additionally, a series analyzing pancreatic precancerous lesions (pancreatic intraepithelial neoplasia) and a pancreatic cancer survey (93) also indicated that Cav-1 may be a good candidate prognostic marker, combined with the upregulation of fatty acid synthase.

#### Renal cancer

The clinical prognostic value of the upregulation of Cav-1 in renal cell carcinoma (RCC) has been clarified. Campbell *et al* (94) were the first to interpret a correlation between the cytoplasmic expression of Cav-1 and the outcome of RCC. The ICC scoring is determined as follows: 0, no detectable deposit in tumor cells; 1, extremely light diffuse or focal light deposit in tumor cell cytoplasm; 2, light diffuse or moderate focal deposit (but may include small areas of heavy deposit); and 3, tumor containing areas of heavy deposit in tumor cells. Among 114 consecutive non-metastatic RCC samples, 50 tumors exhibited ICC scores of 1, 43 of score 2 and 21 of score 3. Statistical analysis revealed that significantly higher scores combined with larger and higher grade tumors, as well as tumors with vascular invasion and Cav ICC scores are independent predictors of poor disease-free survival (82,94,95). Other study has also demonstrated that tumors with upregulated Cav-1 exhibit a positive correlation with tumor diameter and tumor grade/stage (pTNM and pM stages) (96,97). Increased levels of cytoplasmic Cav-1 (P=0.037) have also been clarified to correlate with hematogenous metastasis (98). Survival analysis has independently shown that patients with tumors with increased Cav-1 staining exhibit a shorter overall survival rate (99). Waalkes *et al* (89) initially confirmed that Cav-1 mRNA expression is significantly increased in normal renal tissue (P=0.0003), clear cell RCC (P=1.48×10^−7^) and advanced disease (P=0.019), compared with patients with distant metastasis at the time of diagnosis (P=0.0058).

#### Liver cancer

To date, the clinicopathological role of Cav-1 expression in HCC remains contradictory. Certain reports have demonstrated that the expression of Cav-1 is markedly upregulated in HCC patients (85,86) or cell lines (86). In addition, a marked increase of Cav-1 expression has been identified in metastatic HCC cell lines and tumors compared with normal liver cell lines and all non-tumorous liver tissues. Following the analysis of a cohort of HCC samples, Tang *et al* (85) identified a positive correlation between the upregulation of tumor Cav-1 and the histological differentiation, portal or hepatic venous invasion, intrahepatic metastases and recurrence of HCC. In addition, Cav-1 expression has been found to positively correlate with VEGF expression, microvessel density, and unpaired artery (99). Furthermore, Tse *et al* (86) identified that the overexpression of Cav-1 promotes the growth, motility and invasiveness, as well as tumorigenicity of HCC cells *in vivo.* Similar findings have also been observed in metastatic HCC cells with knockdown of Cav-1. By contrast, studies have suggested that the upregulation of Cav-1 in HCC may serve as a tumor suppressor (75,76). Yan *et al* (75) found that the expression levels of Cav-1 in HCC tissues were significantly lower than those of the adjacent non-cancerous tissues (P=0.026), and that the low expression of Cav-1 is associated with a poor prognosis of HCC.

#### Lung cancer

A positive correlation exists between the upregulation of Cav-1 and the clinical features of primary lung cancer. Although Cav-1 levels in lung tumor tissues are significantly lower than in tumor-free lung tissues (91,92,100–102), the expression of Cav-1 in lung tumor tissues is markedly higher in patients with lymph node metastasis (92,93,100) and advanced tumor stage (93,100,103). Following the statistical analysis of Cav-1 immunostaining and the clinical data of several primary lung cancer cohorts, the expression of Cav-1 was demonstrated to statistically correlate with poor differentiation, pathological stage and lymph node metastasis, as well as a predicted poor prognosis (104). Furthermore, a multivariate analysis of the Cav-1 ICC results of 95 lung adenocarcinoma specimens by Chao-Chi *et al* (101) suggested that Cav-1 is an independent functional predictor of poor survival in lung adenocarcinoma. In addition, Ho *et al* (105) identified that Cav-1 expression significantly correlates with drug resistance and poor prognosis in advanced non-small cell lung cancer (NSCLC) patients treated with gemcitabine-based chemotherapy, by analyzing the immunostaining of Cav-1 and the clinical response to the chemotherapy of 73 NSCLC (stages IIIB and IV) patients.

#### PC

The correlation between the upregulation of Cav-1 and the clinical characteristics of PC has not been completely clarified. However, a higher incidence of Cav-1 expression has generally been found in patients with poorly differentiated tumors (higher Gleason score), positive surgical margins, high tumor stages (TNM T4), lymph node metastasis and poor tumor prognosis (82,106–109). Satoh *et al* (107) further indicated that, in patients with organ-confined (pT2N0) disease, the positive Cav-1 expression was a significant predictor of disease recurrence following radical prostatectomy. An ICC staining analysis of 189 radical prostatectomy specimens (106) identified positive Cav-1 immunostaining as an independent predictor for time to disease progression (P=0.0186). Yang *et al* (110) found that Cav-1 was overexpressed in 41.7% (15 out of 36 patients) of human high-grade prostatic intraepithelial neoplasia (HGPIN) specimens and further revealed a highly significant correlation between Cav-1 (+) HGPIN and Cav-1 (+) PC. Whereas Steiner *et al* (111) found that the number of caveolae was significantly reduced in LNCaP and PC3 cells (P<0.0001), which implied that the downregulation of Cav-1 occurs with the development of PC, while the downregulation of Cav-1 in PC tissues conversely correlates with pT category (P=0.006) and Gleason score (P=0.041).

In addition, the serum Cav-1 levels of PC patients have also been investigated. Certain studies have shown increased serum Cav-1 levels in patients with poor prognosis (112,113). Langeberg *et al* (114) analyzed two case-control (n=1,458 and 1,351, respectively) studies of PC among males in Washington State, USA; however, no correlation was identified between higher post-treatment serum levels of Cav-1 and the risk of aggressive or adverse PC outcome.

#### GC

The role of Cav-1 expression in GC requires further clarification. The ICC study of 405 GC tissue specimens (84) revealed the upregulation of Cav-1 expression in the non-neoplastic gastric mucosa (not detectable) compared with GC [shown in 22 (5.4%) out of 405 cases] tissue. In addition, the upregulation of Cav-1 expression was found to significantly correlate with advanced pTNM stage (P=0.027) and lymph node metastasis (P=0.018). Furthermore, survival analysis showed that Cav-1 expression is an independent prognostic factor of poor survival (P=0.028). However, Gao *et al* (74) analyzed the expression of Cav-1 in 56 GC, 29 non-cancerous mucosa, 11 intestinal metaplasia and seven atypical hyperplasia specimens. The authors concluded a reverse expression trend of Cav-1; the positive rate of Cav-1 was significantly lower in GC than in non-cancerous mucosa, intestinal metaplasia and atypical hyperplasia (17.9 vs. 84.8, 81.8 and 57.1%, respectively; P<0.05). The decreased expression of Cav-1 in GC was found to significantly correlate with differentiation, advanced GC and lymph node metastases. By contrast, Barresi *et al* (115) demonstrated that the role of Cav-1 in GC is not stage-specific or associated with prognosis, following ICC analysis of the expression of Cav-1 in a series of gastric carcinoma and the adjacent normal gastric mucosa.

#### Breast cancer

Previous studies have not reached a consensus concerning the role of Cav-1 in human breast cancer. Certain reports have insisted the tumor suppressive functions of Cav-1 by knockout of the CAV1 gene in cells with a luminal phenotype (116). In addition, Sagara *et al* (73) quantitatively examined the mRNA levels of CAV1 in 162 cases of breast cancer using real-time polymerase chain reaction. Finally, it has also been identified that reduced CAV1 mRNA levels significantly correlate with increasing tumor size (P=0.041) and negative estrogen receptor (ER) status (P=0.021), even though no significant correlation has been identified with disease-free survival (P=0.520). By contrast, other studies (72,83) have identified a positive correlation between the expression of Cav-1, and high histological grade and lack of steroid hormone receptor positivity [ER and progesterone receptor (PR)], as well as the expression of basal markers (basal cytokeratins, p63 and P-cadherin). Furthermore, Joshi *et al* (82) identified an independent prognostic role of Cav-1 expression in human breast cancer, by the multivariate analysis (Cox regression model) of the Cav-1 immunostaining.

#### Other types of cancer

The clinical value of Cav-1 expression in other types of cancer, including bladder, nasopharynx, oral (76,87), colorectal, esophagus, ovarian (90), bone (117) and cerebral (118) cancer, have also been reported. The ICC analysis of several cohorts of esophageal squamous cell carcinoma samples (range, 47–130 samples) (77,78), has identified that positive Cav-1 immunostaining positively correlates with pathological stage (pT, pN and pM stages) and lymphatic or vein invasion, and predicts a significantly shorter overall survival rate. Notably, no significant correlation has been identified between CAV1 mRNA expression and clinicopathological factors (77). Ruan *et al* (119) statistically analyzed the positive expression rates of Cav-1 in primary and recurrent bladder transitional cell carcinoma (BTCC), and the tumor-free survival times in groups with and without Cav-1 expression. The authors also found that the positive expression of Cav-1 predicts a higher recurrence risk of BTCC and shows a lower disease-free survival rate (120). The role of the upregulation of Cav-1 in nasopharyngeal carcinoma (NPC) has been classified by Du *et al* (121). The Cav-1 expression levels were found to significantly correlate with metastasis (P=0.025), a lower five-year survival rate (P=0.02) and local recurrence (P=0.038). Multivariate Cox regression analysis indicated that the combination of high Cav-1 and CD147 expression is a significant, independent prognostic predictor in patients with NPC (hazard ratio=2.135; P=0.006). Survival analysis of the Cav-1 expression in colon cancer (120 samples) and rectal cancer (131 samples) patients (82) has also identified that Cav-1 expression significantly correlates with distant metastasis in colon cancer and decreased disease-free survival (P=0.005) in rectal cancer. In addition, Rödel *et al* (122) demonstrated that local control rates at five years for patients with tumors showing low Cav-1 expression were significantly improved than for patients with high Cav-1 expression carcinoma cells.

### Possible mechanisms associated with the clinical values of tumor cell Cav-1 expression

#### The role of the tumor Cav-1 gene (CAV1)

Contrasting functions of Cav-1 have been demonstrated; a tumor suppressor function and an oncogenic role. Firstly, several epidemiological studies have revealed a correlation between the Cav-1 gene and the risk of several types of cancer. In addition, a number of case-control studies have revealed a correlation between the polymorphism of Cav-1 (CAV1) T29107A (rs7804372) and the risk of PC (113,123) and NPC (124). These studies have independently obtained parallel results, in which a significant difference exists between PC or NPC and the control groups in the distributions of their genotypes and allelic frequencies in the CAV1 T29107A (rs7804372) polymorphism. However, no significant correlations have been identified between this polymorphism and the clinicopathological characteristics which have been declassified (113). More recently, studies have concentrated on the gene expression of Cav-1 in cancer cells. Syeed *et al* (125) investigated 130 breast cancer samples and demonstrated that the gene encoding Cav-1 is associated with the development and progression of breast cancer. Furthermore, the authors revealed that promoter hypermethylation and the loss of expression of the CAV-1 gene is an important alternative mechanism for the inactivation of CAV-1 leading to complete gene silencing (125). In addition, an animal study has identified that low Cav-1 expression is associated with increased cell proliferation, and ERα expression and reduced apoptosis (126).

#### Metastasis

The role of Cav-1 in cell migration is controversial. Evidence is available indicating that Cav-1 promotes migration in a variety of cells, including fibroblasts, endothelial cells and tumor-derived cell lines. Alternatively, the inhibition of migration has been observed in endothelial, pancreatic carcinoma and metastatic breast cancer cells. In pancreatic cancer cells, the Rho protein (RhoC) has a promoting role in tumor metastasis and growth. Lin *et al* (127) demonstrated that high Cav-1 expression may regulate RhoC activity, thus limiting cell migration and promoting growth. In addition, a reciprocal correlation has been identified between Cav-1 expression and p42/p44 Erk activation with PC cell migration, invasion, RhoC GTPase and p38 MAPK activation. Thomas *et al* (128) further demonstrated the phenocopy effect of Cav-1 depletion and the reduced UMUC-3 lung metastasis of bladder cancer *in vivo*, by treatment with a ROCK inhibitor. Arpaia *et al* (129) also demonstrated that the interaction between Cav-1 and Rho-GTPases (most likely RhoC but not RhoA) promotes metastasis. By regulating the overexpression of an activated form of Stat3, Chiu *et al* (130) revealed that the Cav-1 promoter activity and gene expression were increased, preventing the formation of brain metastases. Furthermore, the pathological analysis of a cohort of head and neck squamous cell carcinoma patients suggested that Cav-1 may have an inhibitory function in tumorigenesis and lung metastasis by regulating integrin β1- and Src-mediated cell-cell and cell-matrix interactions (131).

#### Motility and focal adhesion (FA)

The theory that Cav-1 promotes the motility of tumor cells is well established. Following the transfection of a wild-type CVA1 gene, an NSCLC cell line was found to exhibit an enlarged cell shape with filopodia (132). Cav-1 and Rho/ROCK signaling is known to promote the migration and metastasis of tumor cells by regulating FA dynamics through the tyrosine (Y14) phosphorylation of Cav-1. Joshi *et al* (82) further defined a feedback loop between Rho/ROCK, Src and phosphorylated Cav-1 in tumor cell protrusions. The authors demonstrated that phosphorylated Cav-1 expression stimulates Rho activation, stabilizes FAK association with FAs, and promotes cell migration and invasion. However, increased levels of phosphorylated Cav-1 were also associated with elevated Src kinase and Rho/ROCK signaling. The Src family of kinase inhibitors can also reduce Cav-1 phosphorylation on tyrosine-14 and cell migration *in vitro* (133). The Rh/ROCK signaling pathway has also been identified in pancreatic adenocarcinoma cells. Mark *et al* (134) were the first to demonstrate that FA is dependent on c-Src kinase activation, for which Cav-1 is required, in the CEACAM6-overexpressing PDAC cell line, BxPC3. However, Cantiani *et al* (116) found that c-Src and c-Met tyrosine kinases are activated in osteosarcoma and inhibited with Cav-1 overexpression.

#### Antiapoptosis

Meyer *et al* (135) identified that Cav-1 is a crucial hepatocyte fate determinant for TGF-β effects. The knockdown of Cav-1 was found to markedly reduce TGF-β-mediated AKT phosphorylation and, thus, sensitized primary murine hepatocytes for proapoptotic TGF-β signaling. In further study of the androgen-independent PC DU145 cell line, the colocalization of the α_1A_-adrenoceptor with Cav-1 was observed by electron microscopy (136). These results showed that the agonist stimulation of the α_1A_-adrenoceptor induces resistance to thapsigargin-induced apoptosis and that Cav-1 (caveolae integrity) was necessary for this process. By contrast, Rodriguez *et al* (137) found that augmented Cav-1 expression in cells with low basal levels of proteins, such as COX-2 and PGE2, and COX-2 overexpression or PGE2 supplementation, increases the levels of the inhibitor of apoptosis protein, survivin, by a transcriptional mechanism. In a study on human colon and PC cells, it was suggested that Cav-1 regulates the sensitivity to β-carotene growth-inhibitory and proapoptotic effects (138). The authors found that β-carotene functions as a growth inhibitory agent in Cav-1(+) cells, and that the transfection of Cav-1 in Cav-1(−) cells increases cell sensitivity to β-carotene by inducing apoptosis.

## 4. Conclusion

As a main structural component of caveolae, which are plasma membrane invaginations that are involved in vesicular trafficking and signal transduction events, Cav-1 is important in the modulation of cellular signaling. Advances in understanding the contribution of Cav-1 in cancer progression and the clinical characteristics from stromal and cancer cells are likely to enhance the awareness and acknowledgement of the reciprocal signaling that supports and promotes oncogenesis, tumor differentiation, tumor stage, metastasis and survival. Revealing the essential biological and pathological mechanisms involved has realized the requirement for Cav-1-specific therapeutic strategies.

It is already clear that the expression state of stromal Cav-1 is coincidently downregulated in various types of human cancer, including breast cancer, compared with non-cancerous tissues (Table I), and the mechanisms and clinical role of the deregulation of Cav-1 have been sufficiently demonstrated. Future studies are required to varify the role of Cav-1 in other types of CAFs. However, the expression, clinical roles and associated mechanisms of tumor Cav-1 expression are upregulated or decreased based on different cancer types or different experiments of a same cancer type. Cav-1 may have an oncogenic or tumor suppressor role depending on the cell type; however, further investigation of Cav-1 expression and the possible underlying mechanisms are required. In addition, it must be determined whether correlations exist between Cav-1 expression and tumor stromal and cancer cells, and the mechanisms understood.

Despite a number of contradictory Cav-1 studies, the majority of reports markedly suggest that Cav-1 represents an important cancer cell biomarker in carcinogenesis, differentiation, metastasis and tumor progression, and independently serves as a predictor of overall survival rate. In addition, through interaction with other biological molecules, Cav-1 modulates angiogenesis and correlates with chemotherapeutic resistance. To succeed in establishing novel diagnostic molecular and targeted therapies against Cav-1, high-quality, basic and translational studies are required to further unveil the clinical value of Cav-1 expression in multiple types of cancer and tumor stromal cells.

## Figures and Tables

**Table I:** Comparison of Cav-1 expression between tumor stromal and human tumor cells in the literature.

	Expression of Cav-1, n^a^		
		
Tumor components	Upregulation	Downregulation	Total reference counts, n
Tumor cells	53	10	63
Stromal cells	0	15	15
Total	53	25	78

a Numbers represent reference counts.

Cav-1, caveolin-1.
